# Concerted evolution of male and female display traits in the European corn borer, *Ostrinia nubilalis*

**DOI:** 10.1186/1741-7007-7-10

**Published:** 2009-03-03

**Authors:** Jean-Marc Lassance, Christer Löfstedt

**Affiliations:** 1 Department of Ecology, Lund University, S-22362, Lund, Sweden

## Abstract

**Background:**

Sexual reproduction entails the encounter of the sexes and the multiplicity of rituals is parallel to the diversity of mating systems. Evolutionary mechanisms such as sexual selection and sexual conflict have led to the elaboration of traits to gain attention and favours from potential partners. A paradox exists about how coordinated systems can evolve and diverge when there would seem to be a stabilising selection acting. Moth display traits – pheromones – constitute an advantageous model with which to address questions about the evolution of mating systems in animals. Both males and females can possess pheromones that are involved either in close- or long-range communication. Female and male pheromones appear to have different origins and to be under different evolutionary constraints, thus they might be envisioned as independently evolving traits. We conducted laboratory experiments to explore the role of scents released during courtship by males of the European corn borer, *Ostrinia nubilalis*.

**Results:**

Information provided by the male pheromone appears critical for female acceptance. The composition of this male pheromone varies in an age-dependent manner and females show mating preference towards older males in choice experiments. Furthermore, male signals may allow species discrimination and reinforce reproductive isolation. Finally, we found evidence for a genetic correlation between male and female signals, the evolution of which is best explained by the constraints and opportunities resulting from the sharing of gene products.

**Conclusion:**

In this study we used an integrative approach to characterise the male sex pheromone in a moth. Interestingly, the male chemical signal is analogous to the female signal in that structurally similar compounds are being used by both sexes. Hence, in systems where both sexes possess display traits, the pleiotropy of genes generating the traits could influence the evolutionary trajectories of sexual signals and lead to their divergence, with speciation being the ultimate result.

## Background

Understanding the exact ways in which mate choice is exerted is one of the keys to understanding the selective forces driving the processes of assortative mating, reproductive isolation, population divergence and ultimately speciation [1]. Although chemical signalling is one of the major communication channels in animals, few studies have aimed at depicting the importance of scents such as pheromones in mate choice [2-7]. Pheromone bouquets can provide information not only on species and sex, but also on age [5,8]. Even though evidence for the role of pheromones in mate choice is increasing, the mechanisms are still to a large degree unknown [9]. One of the possible explanations for the evolutionary success of Lepidoptera, the second most species-rich insect order, may lie in the efficiency of their mate-signalling systems. Species-specific sex pheromones emitted by female moths mediate long-range mate attraction of males and play an important role in species recognition [2,5,10,11]. In some species, males also possess pheromones that are released at close-range during courtship [5,11-13]. In contrast to the symplesiomorphic nature of the female signalling traits, the male signalling traits in moths are exceedingly variable and labile, suggesting that they have evolved independently on several occasions [11]. The evolution of the male signals has been suggested to constitute an adaptive response in species in which the probability of interspecific mating mistakes is high [14]. Close-range chemical cues have also been proposed as a trait used by females to assess male quality [3,11,15]. Among all male characteristics seen as possible cues for female mate preference in insects, age is one that has received much attention. Authors have proposed explanations as to why females might make mate choice decisions [16-20], but few studies have addressed which sensory cues mediate female choice.

The moth sexual communication system is often seen as an example of a coordinated communication system under stabilising selection because females producing atypical pheromone blends and males responding to 'off blends' are usually at a disadvantage compared with typical individuals, producing and responding to the 'population norm' [10,21]. Following the asymmetric tracking hypothesis [11], males exert weak selective pressure on conspecific females, and by extension, apply low selective constraints on genes involved in female pheromone biosynthesis. It is noteworthy that male moth pheromones generally consist of volatiles acquired or derived from host plant compounds [3,11,12,15], and thus the pheromone biosynthesis taking place in males normally involves a different enzymatic machinery compared with the *de novo *biosynthesis leading to female sex pheromones [22]. The contention is that male and female pheromone communication systems have evolved independently.

**Figure 1:**
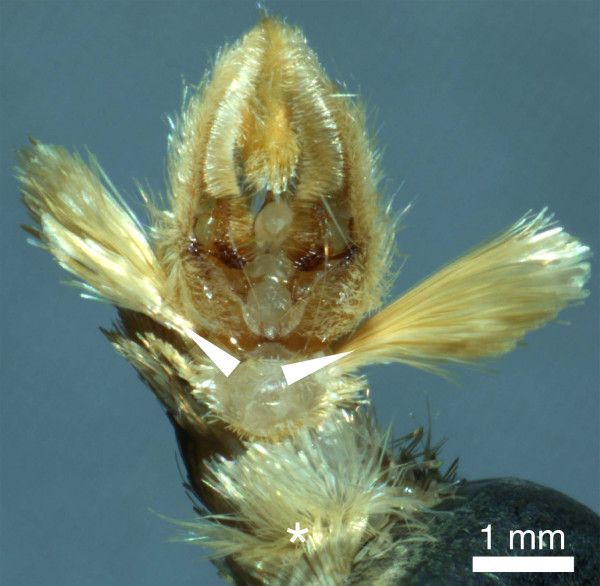
**Hairpencils of corn borer males**. Photograph of the terminal abdominal segments of a male *Ostrinia nubilalis *with hairpencils exposed. One set is located on the 8^th ^sternite (asterisk) while another set is found on the claspers (arrowheads). (Picture JM Lassance).

For various aspects of its ecology and behaviour the European corn borer (ECB), *Ostrinia nubilalis *(Lepidoptera: Crambidae), offers a unique opportunity for studying female choice in Lepidoptera. The majority of females are monandrous [23,24] and male quality has been demonstrated to be of great importance for female reproductive success [25]. As a consequence of their greater parental investment, females can be expected to be selective in their choice of partner and the overall quality of their mates. Indeed, behavioural observations imply that female choice occurs prior to copulation in ECB [26,27]. Male corn borers possess so-called hairpencils located ventrally on the eighth sternite and on the claspers (Fig 1). These organs, normally retracted inside the abdomen, are extruded during the courtship sequence when males are in close vicinity of calling females [26]. In the course of male display, females flick intensively their antennae, suggesting the involvement of male olfactory cues in mate choice, and evidence for male scent to be involved in this mating system has previously been reported [26]. The male pheromone might provide females with a discriminating trait allowing species recognition and/or to assess the overall quality of the males. In addition to the reproductive isolation generated by the male response to female pheromone, the female response to male pheromone would act as a second pre-zygotic barrier reinforcing the reproductive isolation between species. Ethological isolation, caused by female sex pheromone production and perception, is high but not perfect between pheromone strains of the ECB [28-30] and between the ECB and its close relative *O. furnacalis*, the Asian corn borer (ACB). Rare males can be attracted by the pheromone blend of related species or strains, and the formation of hybrids is possible [31-33]. However, the interaction between the so-called Z and E strains of *O. nubilalis *in sympatry results in a lower number of hybrids than one would expect and reveals a high level of assortative mating [34,35]. Crossing experiments revealed the existence of a putative close-range factor determining female preference [36]. Moreover, genetic mapping of the loci causing sexual isolation between pheromone strains of the ECB substantiates the idea that a mechanism different from female sex pheromone prevents gene flow [37]. Comparing the signals produced by males belonging to different species or strains would help to test the hypothesis that male signals promote reproductive isolation.

In many cases, especially when habitat or temporal isolation does not occur, the formation of new species is accompanied by the formation of new pheromones [5,38]. Elucidating how new signals arise is a clue to understanding the generation of diversity. In addition, over the years the ECB and its congeners have become an exceptionally interesting model for the study of evolution of moth pheromones and in particular, the evolution of desaturases, which are key biosynthetic enzymes [38]. Compared with its congeners using Δ11-desaturation in their pheromone biosynthesis to produce (*E*)- and (*Z*)-11-tetradecenyl acetate and (*Z*)-9-tetradecenyl acetate, ACB females use a unique mixture of (*E*)- and (*Z*)-12-tetradecenyl acetate produced through Δ14-desaturation [39]. The discovery of Δ14-desaturase transcripts in ECB females gave rise to many questions regarding the origin of this gene [40,41].

In this article, we report on the nature of chemicals associated with the hairpencils displayed by males prior to mating and their role in female choice. ECB females are able to distinguish between males of different ages and the composition of male scents varies accordingly. Including both ECB pheromone strains and the close relative ACB, we compared the chemical profiles of males and found them to differ significantly. The reconstruction of the putative biosynthetic pathway leading to the production of the male pheromone components highlights analogies between males and females. We found the Δ11-desaturase as well as the unusual Δ14-desaturase to be expressed not only in females but also in ECB males and to be involved in male pheromone biosynthesis. The emergence of the structurally different female sex pheromone used by ACB may thus have been facilitated by activation of genes involved in male pheromone biosynthesis. Our results suggest that the evolution of pheromone biosynthesis genes in corn borers is best explained by a combination of opportunities and constraints that result from their different functions in males and females.

## Results

### Mating experiments

**Figure 2:**
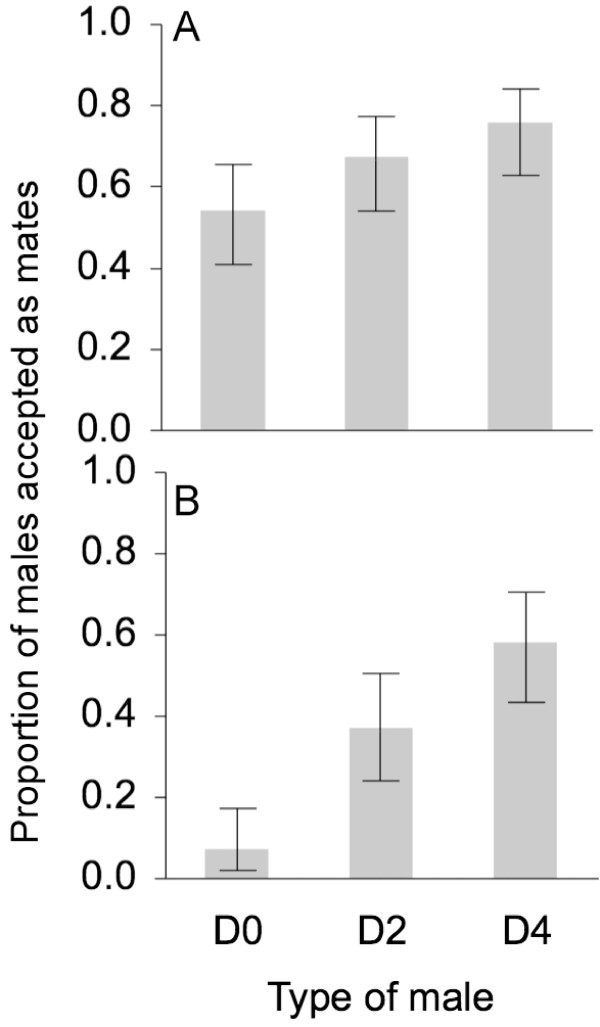
**Male mating success in relation to age**. Proportion of males accepted as mates for each age class when exposed to females in one-male choice experiment (A) or together with males of other classes (B). Overall comparisons reveal significant differences in the proportion of males accepted as mates among age classes (*P *= 0.043 and *P *< 0.001). In both series of experiments females tended to mate more frequently with older males. Error bars depict the 95% confidence limits for the proportions.

We examined whether ECB female mating preference is associated with male age by exposing them to males from three age classes (0-, 2- and 4-day-old males). In a first series of experiments where one female was presented with one male, the proportions of males accepted as mates were significantly different between age classes, females tending to mate more often with older males (χ^2 ^= 6.30, degrees of freedom (df) = 2, *P *= 0.043 for overall comparison (Figure 2A)). No obvious differences were noticed in male courtship display between age classes. In a second set of experiments, where females were given a choice of three males, the overall analysis indicated a significant difference among age classes, with a differential mating success in favour of older males (χ^2 ^= 27.83, df = 2, *P *< 0.001 for overall comparison (Figure 2B)). Males of all classes approached and courted females. Interestingly, females did not necessarily accept the first courting male. Note that in both experiments there was no influence of age on female mating success (χ^2^_exp1 _= 5.42; χ^2^_exp2 _= 0.786; df = 2, *P *> 0.05 for overall comparison).

### Identification of male ECB Z scent

Since we found 4-day-old males to have a higher mating success, we extracted compounds from the hairpencils of individuals of this age. The composition of male ECB Z hairpencils was characterised as a blend of 16 carbon chain-length saturated and monounsaturated acetates. Hexadecanyl acetate (16:OAc) and (*Z*)-9-, (*Z*)-11- and (*Z*)-14-hexadecenyl acetates (Z9-16:OAc, Z11-16:OAc and Z14-16:OAc, respectively) were identified according to their mass spectra and retention times. All the compounds showed the *m/z *61 fragment characteristic of acetates, the ion [M-60], that is, 224 and 222, respectively for the saturated and unsaturated compounds, as well as all other characteristic fragments of the respective compounds. Double bond positions were confirmed by mass spectral analysis of the dimethyl disulphide (DMDS) adducts, which displayed the characteristic ions of hexadecenyl acetates with double bond in the Δ9 (*m*/*z *231, 145, 171), Δ11 (*m*/*z *259, 117, 199) and Δ14 (*m*/*z *301, 75, 241) position, respectively.

### Behavioural assay

**Figure 3:**
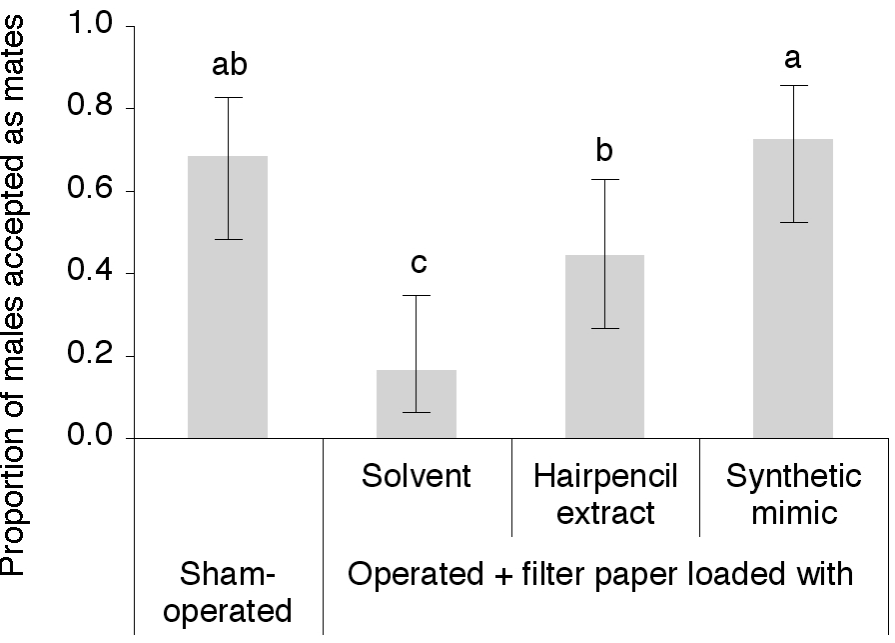
**Behavioural demonstration of the role of hairpencil scents**. Proportion of males accepted as mates by a female exposed to males sham-operated or with ablated hairpencils plus a filter paper loaded with either solvent (hexane), one male equivalent of 4-day-old male hairpencil extract or a synthetic mimic (*N *= 25 males tested for each category). For each category, no letters in common indicate significant differences at the 0.05 level.

A series of experiments was undertaken to verify the behavioural role of the identified compounds. From a previous study [26], we knew that hairpencil ablation has a critical negative impact on male mating success. We hypothesised that male odour was required for females eventually to accept courting males and that female acceptance could be artificially re-established. First, we confirmed that the ablation of hairpencils has a dramatic effect on males' mating success. Female rejection behaviour was observed as wing flapping to maintain the abdomen inaccessible and then by flying away. Operated males obtained significantly fewer mates than sham-operated ones (Figure 3). Second, we were able to re-establish female acceptance by experimentally adding hairpencil odour or a synthetic mimic. This corroborates the importance of the blend of identified hairpencil odorants to female acceptance and male mating success, and qualifies this male-produced volatile blend as a pheromone. However, the activities of the individual compounds of this blend in influencing female acceptance remains to be verified.

### Composition of male ECB Z scent in relation to age

**Figure 4:**
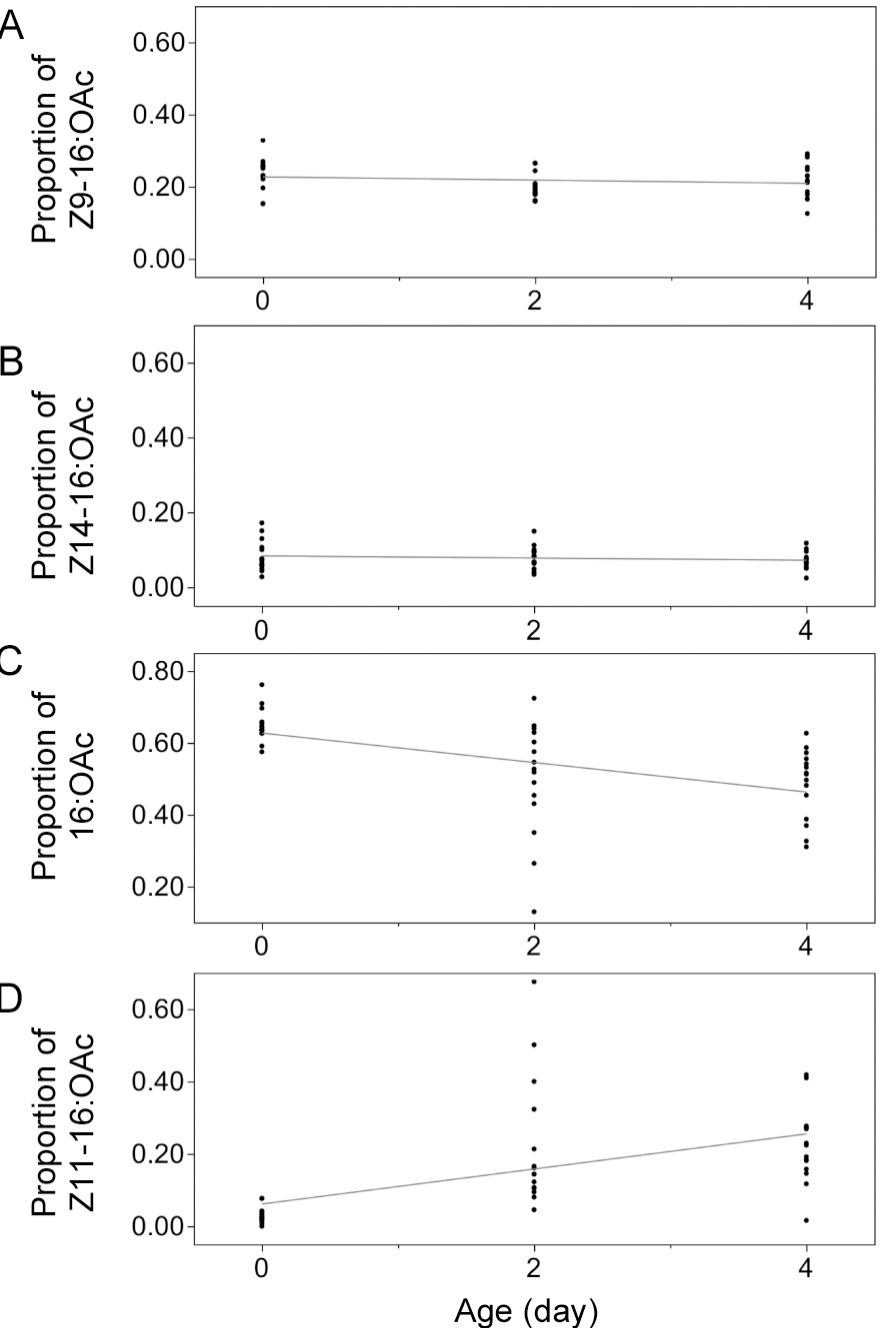
**Relationships between the proportion of individual pheromone components and the age of European corn borer Z males**. The proportion of Z9-16:OAc is not correlated with age (A) (*r*_*s *_= -0.157, *P *= 0.302), neither is the proportion of Z14-16:OAc (B) (*r*_*s *_= -0.054, *P *= 0.722). The proportion of 16:OAc is negatively correlated with age (C) (*r*_*s *_= -0.645, *P *< 0.001), and the proportion of Z11-16:OAc is positively correlated with age (D) (*r*_*s *_= 0.721, *P *< 0.001). *N *= 45 males, 15 per age class.

Differences in male pheromone among age classes could provide a basis for the observed female choice. In some moth species, female preference is correlated with male pheromone content [3,11,15]. Therefore, we regressed the pheromone titre (defined as the sum of the four pheromone compounds) on male age to determine whether females could discriminate males on that basis. Pheromone titre did not correlate with male age (*r*^2 ^= 0.030, *N *= 45, *P *= 0.258; *y *= 2.690 + 0.078*x*; data were natural log transformed), indicating that this trait does not explain the difference in mating success between age classes. However, females could be sensitive to the composition of the bouquet, and thus we tested whether there was a significant correlation between male age and the proportions of the individual components by use of Spearman's rank correlation coefficients (*r*_*s*_) (Figure 4). The proportions of Z11-16:OAc and 16:OAc were significantly correlated with age, positively in the case of Z11-16:OAc, and negatively in the case of 16:OAc (Figure 4C and 4D).

### Comparison of male scent in ECB Z, ECB E and ACB

**Figure 5:**
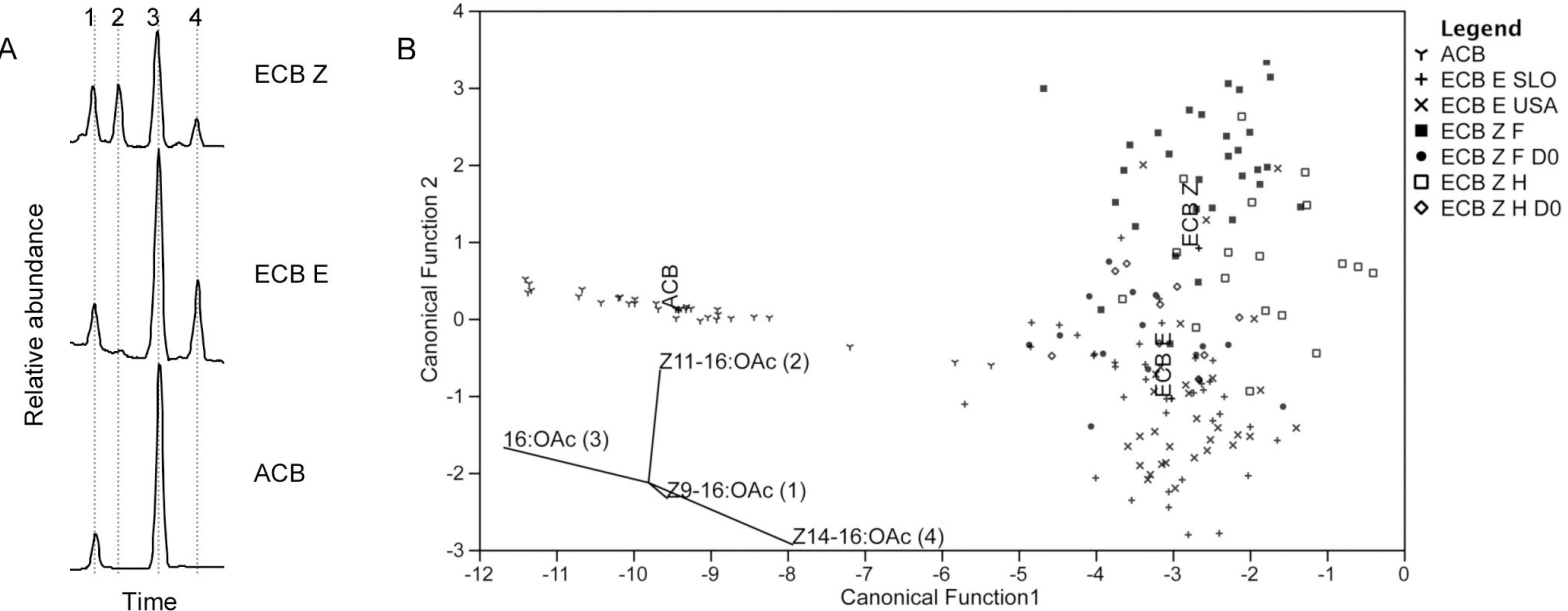
**Composition of male pheromone in corn borers**. (A) Partial gas chromatograms of hairpencil extracts from virgin 4-day-old ECB Z, ECB E and Asian corn borer (1, Z9-16:OAc; 2, Z11-16:OAc; 3, 16:OAc; 4, Z14-16:OAc). (B) Plot of canonical discriminant function scores for each species or strain for the first two functions accounting for 89.2% and 10.8% of the total variance explained. The four variables were absolute amount of individual compounds, all transformed (*Y *= log_10 _(amount + 0.1)).

We compared hairpencil extracts of ECB Z (French population, the main focus of the present study) with extracts of ECB Z from Hungary and also of the other pheromone strain (ECB E strain from USA and Slovenia) and ACB (China). Gas chromatographic analysis revealed major differences in the composition of the male bouquets (Figure 5A). A discriminant function analysis allowed us to resolve individuals of the three corn borer taxa into three groups (Figure 5B). Male ACB produce a mixture of Z9-16:OAc and 16:OAc only, and are discriminated from ECB by the absence of Z14-16:OAc. ECB E individuals differ typically from ECB Z by the low abundance or complete absence of Z11-16:OAc (mean ± standard error of the mean, $\bar{X}$_ECB E _= 0.36 ± 0.10 ng (*N *= 68), $\bar{X}$_ECB Z _= 3.09 ± 0.44 ng (*N *= 72)). ECB E males producing Z11-16:OAc at levels comparable to ECB Z individuals were rarely found (Figure 5B). As reported above, the blend of young ECB Z (D0) contains a low amount of Z11-16:OAc ($\bar{X}$_ECB Z D0 _= 0.45 ± 0.05 ng (*N *= 24)), generating an overlap with ECB E (Figure 5B) and suggesting an incomplete differentiation of the strains for this trait.

### Reconstruction of the male pheromone biosynthetic pathway

**Figure 6:**
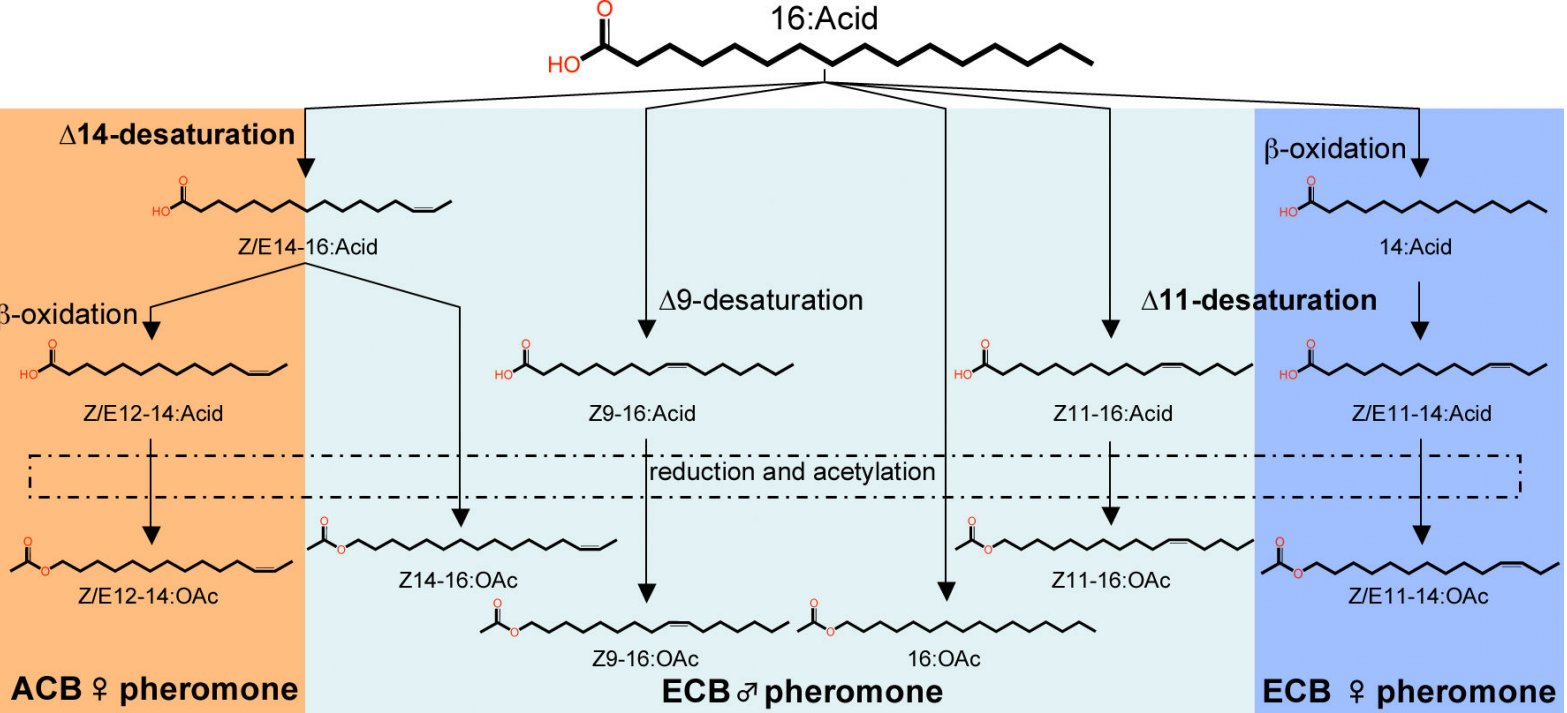
**Proposed pheromone biosynthetic pathways for the European corn borer and Asian corn borer**. The male and female pheromone components in the European corn borer (ECB) and the Asian corn borer (ACB) are biosynthesised *de novo *from the ubiquitous palmitic acid (16:Acid). The routes leading to the 14-carbon acetate components produced by females and to the 16-carbon acetate components used by males employ common key desaturation enzymes. The Δ14-desaturase appears to be common to female ACB and male ECB pathways, as well as the Δ11-desaturase that is shared in male and female ECB pathways. Note that the Δ9-desaturation leading to palmitoleic acid (Z9-16:Acid) also occurs in females of both species, but the reduction and acetylation of this substrate occur in males only. Pathways for male compounds postulated from the analysis of precursors and for female compounds adapted from [40] and references therein.

As male compounds are structurally similar to the ones constituting the female sex pheromone, we hypothesised that the male pheromone components could be synthesised along a biosynthetic pathway similar to those confirmed in female corn borers ([40] and references therein). Our investigations revealed that, in addition to the ubiquitous fatty acids present (including palmitic and palmitoleic acid, likely precursors of 16:OAc and Z9-16:OAc, respectively), the male extracts contained unsaturated fatty acids identified as (*Z*)-11-hexadecenoate supposedly resulting from the activity of a Δ11-desaturase on palmitic acid as well as (*Z*)-and (*E*)-14-hexadecenoate proposed to be produced from the activity of a Δ14-desaturase. Thus, based on precursor analyses and current knowledge on how pheromones consisting of fatty acid derivatives are produced, we postulated the biosynthetic pathways in Figure 6 leading to male pheromone compounds. This suggests that both sexes could rely on the same Δ11-desaturase to produce their pheromone components, as this desaturase can introduce double bonds in carbon chains of different length [40]. Also, a Δ14-desaturase of central importance in the pheromone biosynthetic pathway of female ACB may be part of the male biochemical machinery.

### Detection of Δ14- and Δ11-desaturase transcripts using reverse transcriptase polymerase chain reaction

**Figure 7:**
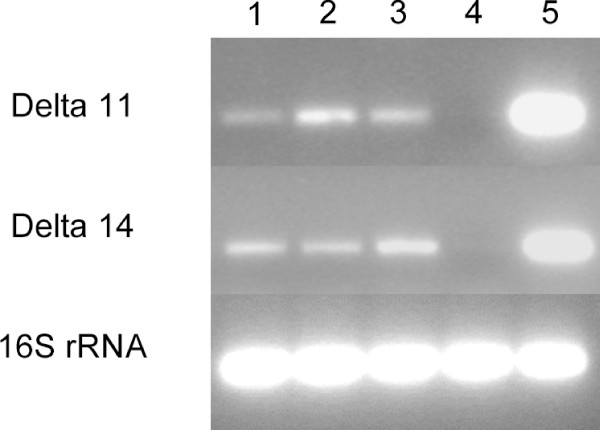
**Reverse transcriptase polymerase chain reaction of Δ11- and Δ14-desaturase transcripts from adult European corn borer tissues**. 1, 2 and 3: abdominal tip from 0-, 2- and 4-day-old males, respectively; 4: abdomen (terminal segments excluded) from newly emerged females; 5: pheromone gland from newly emerged females. Expression of 16S rRNA indicated as control.

Using gene-specific primers, we analysed by reverse transcriptase polymerase chain reaction (RT-PCR) Δ14- and Δ11-desaturase transcript levels in abdominal tips from 0-, 2- and 4-day-old ECB Z males to confirm the involvement of these enzymes in the male pheromone biosynthesis. The expression of both types of desaturase genes could be detected in male tissues, regardless of age (Figure 7). We also analysed pheromone glands as well as abdomens from newly emerged females. As previously reported [40], the results showed expression of the Δ14- and Δ11-desaturase within the pheromone gland but not in the abdomen (Figure 7). However, it was surprising to find that levels of the two desaturase-encoding transcripts were similarly high. Fatty acid methyl ester extracts from ECB Z female pheromone glands were prepared by base methanolysis and analysed by gas chromatography-mass spectrometry (GC-MS). In addition to the fatty acids common to cell membranes and those resulting from the Δ11-desaturase activity, the extracts contained unsaturated fatty acids produced by the Δ14-desaturase (*m*/*z *74, 87, 236 [M-32]). Double bond positions were confirmed with DMDS derivatisation, which produced adducts showing characteristic major ions of Δ14-hexadecenoate (*m*/*z *287, 255, 75) as well as Δ12-tetradecenoate (*m*/*z *259, 227, 75). Taken together, these results show that Δ14- and Δ11-desaturase genes are transcribed, and their mRNAs effectively translated, in both ECB Z males and females.

### Cloning of Δ14- and Δ11-desaturases

To validate the assumption that male and female pheromones are synthesised via shared biochemical pathways, we characterised the Δ14- and Δ11-desaturases expressed in males. We used specific primers designed after the published Δ14 and Δ11-desaturase sequences characterised from female pheromone glands [40] to clone the corresponding cDNAs in males. The Δ14-desaturase transcript possessed nearly identical nucleotide sequence (uncorrected *p *distance (*p*) = 0.0034 and 0.0054 for overall and open reading frame (ORF) comparisons, respectively) and displayed a deduced amino acid sequence identical to the one found in the female pheromone gland. The Δ11-desaturase transcript was more different at the nucleotide level (*P *= 0.0329 and 0.0192 for overall and ORF comparisons, respectively), which corresponds to a deduced amino acid sequence similar at 98.2% to the sequence previously reported for the female pheromone gland. Since these differences could be the result of difference in insect origin (USA previously versus France in the present study) rather than attributable to PCR errors, we cloned Δ14- and Δ11-desaturase cDNAs from pheromone glands of females obtained from our laboratory culture. When comparing the nucleotide and deduced amino acid sequences of male and female clones, we found that the male clones are identical to the corresponding female clones. The sequences reported in this paper have been deposited in the GenBank database [accession nos. EU350083–EU350086].

## Discussion

### Male pheromone as a display trait determining mating success

In lepidopterans and birds males are the homogametic sex. This particular feature has been suggested to render these groups of organisms especially conducive to sexual selection and, therefore, to have favoured the elaboration of male secondary sexual characters [42]. Long tails and bright colours generally come to mind but pheromone signals of male moths are certainly equally elaborate display traits [43]. Behavioural observations throughout the reported experiments provide evidence for the ability of ECB females to distinguish between males, and that male pheromone is a secondary sexual trait associated with female mating preference. In our laboratory setting, male mating success is related to age. Although we cannot dismiss the potential existence of intrinsic differences in aggressiveness and persistence between the age classes, which has been demonstrated in butterflies [44], the results of our experiments demonstrate that there is a correlation between the age of a male and the composition of his pheromone. We propose that female mating preference in ECB is associated with the odour bouquet released by males. A role of male pheromone also in male-male interactions cannot be excluded but was beyond the scope of this study.

Our demonstration of pheromone-based female mate choice and identification of a male-produced courtship pheromone in ECB is of particular importance because it may alter our understanding of the role of pheromones in corn borer speciation. The comparison of male pheromones of the three corn borer taxa indicates a potential species-specific function of these male pheromones. Differences in female sex pheromone between the E and Z strains of ECB have been insufficient to establish a perfect reproductive barrier between these siblings. Pheromones of ECB Z and E malesdiffer noticeably in the presence or absence of Z11-16:OAc. Therefore, male pheromone could act to reinforce reproductive isolation. The evolution of mate choice in ECB Z females may have been partly driven by this difference, since choosing (old) males whose blend contains Z11-16:OAc would result in less hybrid mating. Obviously, future investigations using more populations will be required to elucidate fully this issue. In addition, our results suggest that the male signal could act as an indicator of genetic and/or resource benefits and provide females with information on the quality of the courting males within populations. Thus, female mating preference oriented towards the oldest male in our experiments would be adaptive, females preferring these males because they have proven survival capabilities and may possess a fit genotype for their environment.

### The consequences of sharing gene products on evolution of the display trait

The male corn borer pheromone components are structurally similar to those produced by females and their biosynthesis appears to involve enzymes common to both sexes (Figures 6 and 7). This finding has important consequences for our current view of the evolution of moth pheromones and communication systems in general. First, it may be that genes expressed to generate signalling traits in both sexes have increased functional constraints at a greater number of sites. For example, functional constraints might have arisen on the Δ11-desaturase that was demonstrated [40] to produce a mixture of (*Z*)-11- and (*E*)-11-tetradecenoic acids, which is used in biosynthesis of the female sex pheromone, as well as (*Z*)-11-hexadecenoic acid, which is used in males (Figure 6). Evidence from previous studies suggests that breadth of gene expression and degree of specialisation influence evolutionary rate [45,46]. Indeed, the Δ11-desaturase gene was found to have a slower evolutionary rate compared with the more specialised Δ14-desaturase gene [38]. Also, the sharing of the Δ11-desaturase gene to produce (*Z*)-11-hexadecenoic acid, which is relevant in a male context, and (*Z*)-11- and (*E*)-11-tetradecenoic acids, precursors of the female sex pheromone, may cause antagonistic selection on the enzyme to generate only the type of substrate used in the context of a certain sex. Intralocus sexual conflict will potentially result since the two sexes are under disruptive selection upon a shared character but are genetically constrained from evolving in different directions [47]. Hence, pheromone signals, such as several other display traits, are often found with associated structures that may have evolved through sexual conflict of interest [43]. The sexually antagonistic coevolution driven by this intralocus conflict could be resolved by the evolution of sex limitation, that is, the expression of the enzyme by one sex only, or by the evolution of insensitivity of the female to the male stimulus [48]. This would, however, lead the females into a 'sensory trap' from which it is difficult for them to escape, because if the Δ11-desaturase is not involved in the biosynthesis of the male pheromone or if females evolve insensitivity, they also lose the naturally selected benefit of their preference as Z11-16:OAc allows them to distinguish between age classes and/or ensures the distinction between Z and E males.

The existence of a genetic correlation between male and female signals can also be the source of evolutionary opportunities. Genes implicated in one sex's trait could serve in the context of the other sex's trait, leading to the emergence of a new signal. The presence of individuals responding to this new signal could help its maintenance and generates suitable conditions for divergence and possibly speciation to occur. Our results suggest an answer to the intriguing question as to why a sex pheromone biosynthesis gene that does not function to produce, for instance, the ECB female sex pheromone, would be maintained in the genome over long-term evolutionary time [40,41]. Our findings imply that the Δ14-desaturase gene in question is functional in males and involved in the biosynthesis of a putative male pheromone component (Z14-16:OAc) (Figure 6). It can be speculated that the processing of the Δ14 gene as a new functional desaturase gene occurred in males of an ancestral *Ostrinia *species. Members of the same gene subfamily were found in the *Drosophila melanogaster *genome, suggesting that these genes have derived from a duplication event that took place before the split between Diptera and Lepidoptera [40]. However, the presence of Δ14-desaturase or its products have only been reported in the lepidopteran genus *Ostrinia*. It is tempting to place such rarity in parallel with the polyphyletic nature of male-pheromone systems [11]. Interestingly, in addition to the expected Δ11 transcripts, a high level of Δ14-desaturase transcripts was noticed in the female pheromone gland. Firm evidence for the enzyme to be active in that tissue was brought by the detection of fatty acids unsaturated at the fourteenth position. Trace amounts of Δ12-tetradecenoic acids were also detected in the pheromone gland of the ECB Z female. Produced through one step of limited β-oxidation, these acids constitute the precursors for the female pheromone of the ACB [49] (Figure 6). This suggests that the differences between ECB Z male and female pheromones may not be the result of differential activity of the desaturase systems but rather of differences at other steps in the biosynthesis. A major shift in the expression pattern is likely to have occurred in an ancestral *Ostrinia *population and given rise to the ACB [40,41]. Under such a scenario, some females would have started to emit a pheromone blend consisting of a mixture of Δ11- and Δ12-tetradecenyl acetates [50]. The emergence of the new blend, and subsequent tracking by responsive males, would have been facilitated by the pre-existence of enzymes linked to a biosynthetic pathway involving Δ14-desaturation and olfactory receptors devoted to the detection of Z14-16:OAc. Our results thus identify a mechanism that facilitates the rapid evolution of pheromones by large saltational shifts. Parallel to the pleiotropy of genes producing the signal may be the pleiotropy of genes responsible for signal detection and interpretation. Investigations are under way to determine the existence of a link between male and female pheromone reception.

## Conclusion

One of the conundrums for evolutionary biologists is how coordinated communication systems under stabilising selection can still diverge and lead to the process of speciation [1,5]. The revealed diversity of male pheromone systems [12] is likely to be parallel to diversity in genes generating the associated traits. Indeed, what we describe above could have occurred in other genera or families and, therefore, have significantly influenced the evolution of female pheromone production in many lepidopterans. Additionally, our findings show how new sexual traits could emerge from changes in expression pattern and set the scene for speciation. Further studies should aim at elucidating the mechanisms that generate such changes and how new traits can become fixed.

## Methods

### Moths

Insects were obtained from laboratory cultures reared on a maize-wheat germ diet. Eggs or pupae from the different strains were obtained from: ECB Z, France (French National Institute for Agricultural Research (INRA), UE Entomologie, Poitou-Charentes, France) and Hungary (T Dekker, Swedish University of Agricultural Sciences (SLU), Alnarp, Sweden); ECB E, USA (WL Roelofs, Cornell University, Ithaca, USA) and Slovenia (T Dekker, SLU); ACB, China (CH Zhao, Academia Sinica). Males and females were separated before eclosion and placed in different climate chambers maintained at 23 ± 1°C in a 17-hour:7-hour light-dark photoperiod. Newly emerged adults were separated daily and considered to be 0-day-old.

### Mating experiments

We compared the mating success of males in relation to age. To test this we defined three age classes (0-, 2-, 4-day-old males) and set up two experiments in which females were in the presence of either one or three males of each class. ECB Z males and females were placed together into a cylindrical container (1 litre) with a source of water 1 hour before the onset of scotophase. After the onset of scotophase and during the entire 7-hour scotophase, events in the mating enclosure were monitored at intervals of 15 minutes in order to check courtship behaviour and mating occurrence. In the first series of experiments, we subjected each female to a one-male choice test (*N *= 60 individuals for each of the three male classes, giving a total of 180 females), scoring the formation of mating pairs. For each age class we calculated the proportion of males accepted as mates (number of pairs observed/number of males tested). A χ^2 ^test was used to test the dependence of male mating success on age. In the second set of experiments, 60 females were permitted a choice between three males, one from each age class. Each male was anaesthetised and marked on the thorax using a paint marker pen and given a colour corresponding to his age class. Colours were rotated between trials and had no detectable effect on male mating success. For each mating pair observed (*N *= 47), we noted the age class of the male. The proportion of males accepted as mates for each age class was calculated as the number of males of a given class accepted as mates over the number of mating pairs observed. Again, a χ^2 ^test was used to test the dependence of male mating success on age. Equal numbers of 0-, 2- or 4-day old females were used in the experimental design of both experiments. The 95% confidence intervals of individual proportion were computed according to the method described by Newcombe [51] using the online tool available at Vassarstats [52].

### Identification of male ECB Z scent

Volatiles were extracted by placing hairpencils in hexane and recovering the solvent after 1 hour at room temperature. The samples were analysed on a GC (Hewlett-Packard 5890) connected to a MS (Hewlett-Packard 5972, EI 70 eV). A HP-1MS column (methyl siloxane, 30 × 0.25 mm, df = 0.25 μm, Agilent Technologies) was used and the oven temperature was maintained at 50°C for 2 minutes and then programmed at 10°C per minute to 250°C kept for 10 minutes (carrier gas helium, velocity 30 cm/second). The compounds were identified by comparison of their spectra with standard mass spectra and retention times. Confirmation of double bond position was obtained by DMDS derivatisation and subsequent GC-MS analysis [53].

### Behavioural assay

In a one-male choice assay, we exposed females to 4-day-old males from which hairpencils had been ablated surgically on the day preceding the experiments. In order to facilitate hairpencil ablation, males were anaesthetised with carbon dioxide. Hairpencils were extruded by applying gentle pressure on the abdomen and trimmed with fine forceps to remove as much as possible. The same procedure was used on the sham-operated males with no removal of the hairpencils. The assays were conducted in a chamber consisting of a glass cylinder (13 cm diameter × 25 cm height) with steel screening covering the open ends. Airflow was generated by placing the arena in a wind tunnel together with the addition of a small fan. First, one calling female moth (0- to 4-day-old) with the pheromone gland exposed was introduced into the arena and placed upwind from the males. Single 4-day-old virgin males were then added downwind. Each male was given 10 minutes to mate successfully with the female. Odour replacements were made by introducing an odour source (filter paper) upwind from the female while the male was courting. The odour source was loaded with either 4-day-old male hairpencil extract (one male equivalent) or a synthetic blend mimicking the odour of those males. The synthetic mimic consisted of a blend of 20% Z9-16:OAc, 15% Z11-16:OAc, 53.5% 16:OAc and 11.5% Z14-16:OAc corresponding to 4/3/11/2 ng of individual compound in 20 μl of hexane. Positive and negative controls consisted of sham-operated males and operated males plus a filter paper with the solvent alone applied on to it. The trial was ended either when successful coupling was observed (the male was considered 'accepted as mate') or at the end of the allotted time. Each treatment was tested using 25 males. All the males included in the statistical analysis displayed and attempted to copulate with females. The proportions of males accepted as a mate in individual treatments were compared using *z*-tests (α = 0.05).

### Composition of male scent in relation to age and taxa

To characterise hairpencil pheromone titre and composition in relation to age (ECB Z) and taxa (ECB Z and E and ACB), hairpencil volatiles were extracted in heptane containing a known amount of pentadecanyl acetate chosen as internal standard. Samples were analysed on a GC (Hewlett-Packard 5890) equipped with a flame ionisation detector. A HP-1MS column was used and the oven temperature was maintained at 80°C for 2 minutes and then programmed at 10°C per minute to 250°C kept for 10 minutes (carrier gas helium, velocity 60 cm/second).

### Statistical analyses

All statistical tests were carried out in SPSS 16 software with the exception of the multivariate analysis of variation in pheromone components (absolute amounts in ng) of ECB Z and E and ACB males performed by canonical discriminant function analysis using JMP 7 software.

### Precursor analysis

Fatty acid methyl ester extracts were prepared by base methanolysis. Total lipid extraction was performed by immersing ECB Z male abdominal tips in 100 μl of chloroform:methanol (2:1 v/v). After 1 hour, the tissues were removed and a gentle stream of nitrogen was applied to evaporate the solvent. Conversion of fatty acyl moieties to methyl esters was made by treating the samples with 100 μl of potassium hydroxide (0.5 M in methanol). The reaction was ended after 1 hour by the addition of 100 μl of hydrochloric acid (1.0 M in water). The fatty acyl methyl esters were recovered in hexane and the samples subsequently analysed by GC-MS. Double bond positions were confirmed by DMDS derivatisation.

### Collection of insect tissue and RNA extraction

Male abdominal tips were carefully dissected from 0-, 2- and 4-day-old virgin male moths and stored at -80°C. Pheromone glands and abdomens from 0-day-old virgin female moths were dissected and stored similarly. Total RNA was isolated and purified from dissected tissues using the TRIzol reagent (Invitrogen) according to the manufacturer's recommended procedures.

### Cloning and sequence analysis of Δ14- and Δ11-desaturases from male and female

Based on the publicly available sequence information (Δ11: AF441221; Δ14: AF441220), specific primers were designed to obtain sequence information corresponding to the Δ14- and Δ11-desaturases expressed in male and female *O. nubilalis *Z (primer sequences reported in Table 1).

**Table 1:** Primers used for polymerase chain reaction and reverse transcriptase polymerase chain reaction amplification.

**Gene**	**Primer**	**Sequence (5' to 3')**
*Δ14*	Δ14-ORF-s	CCCAGCAAACATGGCAGACATAGAC
	Δ14-ORF-as	CGCGAAACTTCTACGCACCGTAACTT
	Δ14-5'RACE	CGCTCATGTCAATGGTCTTCCTCAGCTT
	Δ14-3'RACE	ATGAACAAAGGAGACGGCACTTACGAGG

*Δ11*	Δ11-5'RACE	GCCAAGCACAGCAAGATTCAGAAGAACG
	Δ11-3'RACE	GATTGGAATCACTGCTGGTGCCCATAGA
	Δ11-RT-s	GGCATTTCAAAATACTGCGCTCTCGTGG

*16S rRNA*	16S-s	TGAAGGGCTGCAGTATTTTG
	16S-as	TCGAGGTCGCAAACTCTTTT

Total RNA (100 ng) was used to amplify a fragment corresponding to the ORF of the Δ14-desaturase gene. Two primers (Δ14-ORF-s plus Δ14-ORF-as) were used with the Superscript III One-Step RT-PCR kit (Invitrogen) following the manufacturer's instructions. Amplification products were analysed by electrophoresis on agarose gel. An amplification product of around 1250 base pairs was excised from the gel, purified using the Qiagen gel extraction kit (Qiagen) and cloned using TOPO TA cloning kit with PCR2.1-TOPO vector and One Shot TOP10 chemically competent *Escherichia coli *(Invitrogen) for sequencing. Subsequently, primers were designed to obtain 5'- and 3'-ends by rapid amplification of cDNA ends (Δ14-5'RACE and Δ14-3'RACE, respectively). We used the SMART RACE cDNA amplification kit (Clontech) following the manufacturer's protocol. The coding sequences were deduced with the sequencing result of the 5'-end, central region and 3'-end.

For the Δ11-desaturase, two primers were designed to generate overlapping amplification products by rapid amplification of cDNA ends (Δ11-5'RACE for the 5'-region and Δ11-3'RACE for the 3'-region). The coding sequences were deduced with the sequencing result of the 5'- and 3'-ends.

### RT-PCR

Total RNAs (50 ng) were used to amplify fragments of the Δ14- and Δ11-desaturase genes using the Superscript III One-Step RT-PCR kit (Invitrogen) following the manufacturer's instructions. The gene-specific primer sets used were Δ11-RT-s and Δ11-5'RACE for Δ11, Δ14-ORF-s and Δ14-5'RACE for Δ14. PCR products were analysed on a 1% agarose gel and visualised with ethidium bromide.

## Abbreviations

16:OAc: hexadecanyl acetate; ACB: Asian corn borer; df: degrees of freedom; DMDS: dimethyl disulphide; ECB: European corn borer; GC: gas chromatography; MS: mass spectroscopy; ORF: open reading frame; RT-PCR: reverse transcriptase polymerase chain reaction; Z9-16:OAc: Z11-16:OAc, Z14-16:OAc, (*Z*)-9-, (*Z*)-11- and (*Z*)-14-hexadecenyl acetate, respectively.

## Authors' contributions

JML participated in the design of the study, carried out the experiments, performed statistical analyses and drafted the manuscript. CL financed, mentored and participated in the design of the study and helped to draft the manuscript. Both authors read and approved the final manuscript.
